# “I want to really crack this nut”: an analysis of parent-perceived policy needs surrounding food allergy

**DOI:** 10.1186/s12889-020-09309-w

**Published:** 2020-08-01

**Authors:** Elissa M. Abrams, Elinor Simons, Jennifer Gerdts, Orla Nazarko, Beatrice Povolo, Jennifer L. P. Protudjer

**Affiliations:** 1 Section of Allergy and Immunology, Department of Pediatrics and Child Health, University of Manitoba, Winnipeg, MB Canada; 2 Food Allergy Canada, Toronto, ON Canada; 3 Participant Advisory Committee to J Protudjer, Winnipeg, MB Canada; 4 Advocacy and Media Relations, Food Allergy Canada, Toronto, ON Canada; 5 University of Manitoba, 501G-715 McDermot Avenue, Winnipeg, MB R3E 3P4 Canada

**Keywords:** Children, Food allergy, Patient-oriented research, Perceived needs, Policy, Qualitative

## Abstract

**Background:**

In Canada, anaphylaxis-level food allergy constitutes a legal disability. Yet, no nationwide policies exist to support families. We sought to understand what parents of children with food allergy perceive as the most pressing food allergy-related policy concerns in Canada.

**Methods:**

Between March–June 2019, we interviewed 23 families whose food allergic children (*N* = 28mean age 7.9 years) attending an allergy clinic in Winnipeg, Canada. Interviews were audio-recorded, transcribed and analyzed using content analysis.

**Results:**

Over 40% of children had multiple food allergies, representing most of Health Canada’s priority allergens. We identified four themes: (1) High prevalence. High priority?. (2) Food labels can be misleading, (3) Costs and creative ideas, and (4) *Do we have to just deal with the status quo around allergies?*

**Conclusion:**

Food allergy ought to be a national policy priority, to improve the process for precautionary labelling, to improve funding, educational tools access to care, and knowledge of current allergy guidelines.

## Background

In high-income nations, the estimated prevalence of probable pediatric Immunoglobulin E (IgE)-mediated food allergy ranges from a low of 1.9% in Iceland [1], to 6.6% in Australia [2]. Estimates from Canada fall at the higher end of this range, at 6.7% [3]. Over the past decades, food allergy prevalence has increased dramatically,. although a recent report suggests that this level, although at an all-time high, has reached a plateau [3]. Food allergy has a significant impact on day-to-day health-related quality of life of affected children [4–6] and their families, and contributes to increased child and parental anxiety [7, 8]. Moreover, there have been reported incidences of food allergy-related bullying [7, 8]. In fact, in some studies food allergy has a more significant impact on quality of life than other chronic diseases of childhood [7, 9].

Whereas there has been a burst in understanding about the pathophysiology, diagnosis, and treatment of food allergy, little literature exists on the expectations and priorities of some of the most important stakeholders in food allergy research – the families of food allergic children. The United Kingdom [10], Canada [11], Australia and New Zealand [12] and the European Union [13], have detailed guidelines to support those living with food allergy. In many of these regions, allergy-related food recalls remain high [14], as many as 30% of products have undergone risk assessment screening, yet carry no precautionary allergen labelling (PAL) [15], and consumers remain unclear about PAL [14, 16]. Yet, while patients’ voices are critical in food allergy research [17], little is known about how those directly affected by food allergy perceive current guidelines and gaps. Our team of patients, patient advocates, scientists and clinicians sought to address this knowledge gap in Canada. Specifically, we aimed to identify what parents of children with food allergy perceive as the most pressing food allergy-related policy concerns.

## Methods

Parents of children with pediatric allergist-diagnosed food allergy were recruited from a tertiary care allergy clinic in Winnipeg, Canada, between March and July 2019. This clinic services a catchment area of over 1.5 million people from Manitoba, Nunavut and Northwestern Ontario (Manitoba). Pediatric IgE-mediated food allergy was diagnosed based on either positive skin prick tests (wheal > 3 mm) to a particular food, in combination with a convincing clinical history of an immediate allergic reaction to the same food, and/or a failed oral food challenge (OFC).

When parents and their children presented to clinic for a routine follow up or OFC, a trained research assistant approached families to invite them to participate in an interview study on IgE-mediated food allergy. If families provided written consent, a mutually convenient time was coordinated at which families participated in an interview led by a research team member.

One-on-one interviews were conducted in a quiet area (*n* = 24) or by telephone (*n* = 4), per participants’ preference. Interviews began with discussions around parental mental health, and social and financial impacts of food allergy, and culminated in discussions of perceived needs surrounding food allergy-related policy. Following a semi-structured interview guide developed specifically for this study (see Additional File 1), parents were asked what they would like policy makers at any level to know about food allergy, and what types of supports would be most beneficial. Interviews averaged 49 (range 23–68) minutes, and were recorded with written parental consent. Interviews were transcribed verbatim.

Data were analyzed using thematic analysis [18]. This two-step process involved reading the transcripts for surface descriptive content, and organising common ideas, the re-reading the transcripts to look for latent, or underlying meaning to comprehensively understand what parents described. As part of this analysis process, we iteratively developed and systematically applied a coding guide. Subsequently, latent content, or larger themes, were critically developed and applied to the data. To enhance rigour through member-checking [18], representatives from our national patient advocacy organization, Food Allergy Canada, and our local parent advisory council were actively involved at all stages of data analysis and interpretation.

Once no new or additional constructs were identified with subsequent interviews, constructs were deemed to be saturated. Parents received a cinema gift card and coupons for allergy-friendly foods as honoraria. The University of Manitoba Heath Research Ethics Board approved this study.

## Results

In total, 23 interviews were conducted with parents whose children (*N* = 28) had allergies to a variety of foods, including both priority and non-priority allergens. In total, 18 families had 1 food allergic child, 5 had two allergic children, and 12 children had multiple food allergies. Of those children with multiple food allergies, the most common combination was peanut and tree nut (*n* = 4), and *n* = 2 children each had allergies to six foods. Children were, on average, 7.9 (range 0.6–16) years old. The mean age at diagnosis was 2.2 years, indicating that most had lived with their food allergies for many years (Table 1). Mothers typically attended interviews, and nearly all families lived in large, urban centres. The themes below were identified in response to queries as to what type of policies by food allergic families believed would be directly beneficial. The frequency of mention of each of the themes in presented in Table 2.

**Table 1:** Demographic and allergy-related characteristics

	n	%
Characteristics of children with allergies (*N* = 28)		
Age in years (mean; range)	7.9	0.6–16
Age at diagnosis (mean; range)	2.2	
Boys	15	53.6
Multiple food allergic	12	41.4
Food allergies^a^		
Milk	6	21.4
Egg	6	21.4
Peanut	15	53.6
Tree nuts	11	39.3
Fish/Shellfish	5	17.9
Soy, peas, other legumes	3	10.7
Other	1	3.4
Parent characteristics (*N* = 23)		
Mothers	21	91.3
Mother employed	20	87.0
Urban residency	22	95.7

^a^Not mutually exclusive

**Table 2:** Themes and frequency of mention

Theme	Frequency of mention
High prevalence. High priority?	9
Food labels can be misleading	10
Costs and creative ideas	17
*Do we have to just deal with the status quo around allergies?*	8

### Theme 1: high prevalence. High priority?

Although food allergy prevalence is at an all-time high and often discussed in the media, some parents perceived that food allergy remained misunderstood by policy makers and other stakeholders, such as school administration, food producers and *‘politicians*.’ For food allergic families, avoidance is the cornerstone of management. The message delivered at time of diagnosis was that the child must avoid the food(s) to which he/she is allergic, or suffer the consequence of a potentially severe reaction. Although such management may sound straightforward to those who do not have a family member with the disease, parents expressed concern at the *“silent impact,”* or the daily challenges of avoiding certain foods. Parents wanted policy makers to understand that avoidance also has consequences including a constant need for planning and preparedness, the loss of the ability to be spontaneous, continual food allergy education and advocacy at a community level, and uncertainty in eating anything that was not prepared at home. One mother described what she would say if she had 5 minutes with a policy maker:

If I had five minutes, honestly, I would spend the first two, three or even four minutes explaining [food allergy] to them, because a lot of people don’t understand the whole process and everything that we go through*.*

Other families expressed concern that policy makers and government do not recognize the impact of food allergy. Whereas populations often turn to government for support, these families were concerned that their voices were not being heard or “*taken seriously.”*

### Theme 2: food labels can be misleading

Clear and complete disclosure of ingredients on food labels is *“extremely important”* for food allergy management. Whereas families recognized that clear labelling laws may be *“a pain for the food manufacturer,”* nearly all families expressed frustration and concern surrounding overuse of “may contain” on pre- packaged foods. This phrase was perceived as confusing and a means for food manufacturers to protect themselves, rather than being a way for food allergic families to make informed choices. As one mother said, *I really want to crack this nut of may contain! Does it or doesn’t it?! I think we can expect food manufacturers to know [what their products contain].* The lack of clear labels, and legislation that requires allergen labelling of only prepackaged foods makes it difficult for families to make informed choices, which contributed to many families feeling “*limited*” and “*narrowed what [they] can have*.”

Similarly, parents whose children were allergic to some, but not all tree nuts, were concerned that the blanket statement, “*contains tree nuts*” hindered their ability to purchase some products. These parents called for more detailed labels that detail the specific nut(s) in the product so they would not have to exclude all tree nut-containing foods.

Parents also called for government to legislate, rather than the current practice of recommending, that food manufacturers position other precautionary allergen information on a specific place on the food label. All families talked about carefully reading ingredient lists and “may contain” labels, but many were frustrated about the inconsistent placement of information on “processed in a facility” that also handles various allergens. As such, parents described missing this information at time of purchase as “*the most common mistake*” made by families without food allergy.

### Theme 3: costs and creative ideas

In Canada, anaphylaxis-level food allergy now constitutes a legal disability. Yet, at present, there are no nationwide policies in place to support families. All parents commented on the high cost of epinephrine autoinjectors (EAI), and expressed gratitude that they had private or group insurance that substantially offset these costs. But, as one mother said, *“Heaven help the people who are living up north or [who aren’t] middle income.’* Nearly all families called on government and policy makers to impose mandatory insurance coverage and/or tax credits for this life-saving medication. One parent offered an interesting suggestion, in which expired EAI could be *“traded in for a new one and you pay half the price’* for a new one.

Parents also commented on the need for stock epinephrine, using the same model as exist for automated external defibrillators. Stock epinephrine is an epinephrine auto-injector that is not prescribed for a particulate person and may be used in an emergency to help an individual who is having an allergic reaction [19]. Whereas some jurisdictions advertise stock epinephrine in public venues, the lack of a nationwide policy was viewed as “*not acceptable*.” Similarly, some parents were unaware of the legal protection of those who administer, in good faith, a stock EAI to their child.

Nearly all families called for a tax credit to offset the costs of allergy-friendly foods. Food allergy was viewed as “*forcing you to eat healthier… but costs more money…and the choices are limited*.” The dollar amount most families indicated which would be meaningful was relatively modest, *“50 dollars a month or something,”* but which would be adjusted according to the type and number of allergies.

### Theme 4: *Do we have to just deal with the status quo around allergies?*

Current management practices for food allergy involve avoidance of the allergenic food and its derivatives, and often, the prescription of an EAI. The final theme, which is a direct quotation from one mother, reflects the need for continued funding for food allergy research, awareness and support “*as there’s not enough push and money being put into the future of allergies*.”

Many parents expressed gratitude for their child’s allergist, especially those who performed OFC when thought to be clinically appropriate. OFCs involve introducing small, but incrementally increasing doses of a suspect food over time in a controlled environment, to a carefully characterized child to confirm a diagnosis or rule out the possibility of allergy. The clinical decision to perform an OFC is based on a constellation of factors, many – such as IgE levels – are based on outcomes from previous research studies. Parents whose children passed the challenge said that “*it was like some of the research had caught up with my dreams… the dreams that I had for my kid*.”

Allergen avoidance remains the cornerstone of food allergy management. Yet, some families wonder if that was all that could be done. A small group of parents expressed frustration that immunotherapy trials are being conducted internationally, and in other parts of Canada, but not in their communities One family described how their child had been recently been accepted into a potentially life-changing food allergy clinical trial, but had to decline because it was logistically impossible for them.

## Discussion

To our knowledge, this is the first study of its kind to address the changes in policy deemed high priority by the some of the most important stakeholders in food allergy research in Canada – the families of food allergic children. It notes four themes as described by families of food allergic children – the consequences of food avoidance, the barriers imposed by allergen labeling, the cost of living with food allergy, and the need for ongoing funding to address issues surrounding the impact of food allergy.

This study highlights the need for increased policy and resources at the federal level, such as ingredient declarations and allergen labelling, and at the provincial level, including healthcare delivery and education. For example, in 2005, the Canadian province of Ontario (the most populous province in Canada) passed legislation under the Education Act, which requires an anaphylactic policy in schools, and the administration of EAI or other medication by a school employee if a student is suspected to be experiencing anaphylaxis, even if there is no pre-authorization to do so [20]. Similar legislation was passed in the Canadian province of Alberta in 2019 [21]. Supports for allergy management and education also occur at various levels, including at the civic and institutional level. For example, some school divisions outside Ontario and Alberta have passed motions to purchase back-up EAI (also described as stock epi) for students and staff, based on student ratio, who have previously been prescribed such devices [22]. Moreover, our study highlights the need for orchestrated and planned lobbying by the allergen community to financially support the development of appropriate policy and resources. The Australian National Food Allergy Strategy provides an excellent example of how multi-stakeholder partnerships can contribute to improved standards of, and access to care; evidence-based information, education and training; patient-focused research; and recognition of allergic diseases as a prioritized chronic disease [23].

Whereas not all food allergic children require special dietary products, Health Canada requires that most, but not all prepackaged foods, carry a label on which priority allergens are listed [24]. In Canada, mandatory labelling regulations apply to priority allergens, namely milk, egg, peanut, tree nuts, fish, crusteaceans and molluscs., soy, wheat and triticale, sesame, mustard and sulphites [11]. Whereas these are the most common allergens in Canada [3], they are not the only allergens. The notable absence of the requirement to label non-priority allergens in some products may be a source of frustration for families who manage these allergies.

Nearly all families in the study also expressed frustration about PAL on prepackaged products. Manufacturers began including precautionary allergen labeling such as ‘may contain’ in an effort to help consumers avoid food allergens due to a possible risk of cross-contamination. These labels are read up to 99% of the time by food allergic individuals and their families [25]. In a study of 59 Australian food manufacturers, at least 30% of foods that had undergone risk assessment carried no PAL [15]. This is despite Australia’s implementation of Voluntary Incidental Trace Allergen Labelling (VITAL®), a standardized risk assessment tool developed for the food industry to produce “label outcomes” based on a recipe, food source, and risk of cross-contamination [26]. In Canada, PAL statements are not regulated, there is no current requirement for manufacturers to assess or substantiate the use of PAL, and blanket ‘may contain’ statements (such as: This product may contain X priority allergy as well as any other allergens) may be used by some manufacturers in place of implementing manufacturing procedures to address and/or mitigate the risk of cross-contamination. Such voluntary guidance stipulates only that health claims must be truthful and not misleading [11].

It has been noted that the amounts of allergen in a ‘may contain’ label may vary tremendously and hence vigilance is recommended with these products [27]. This may lead to two paradoxical effects. As so many products contain precautionary labeling, many families ignore PALs, which can lead to accidental ingestions and reactions [28]. In fact, 40% of food allergic patients in the United States and Canada purchase food products with PALS [29]. Whereas current regulations call on manufacturers to identify specific types of food allergens (such as almond, or salmon) in the ingredient list, rather than the general term (such as tree nuts or fish, respectively), issues still remain for PALS [11]. In stark contrast, the other risk is unnecessary dietary restrictions of prepackaged foods that do not, in fact, contain the allergen of concern. This is most poignantly a risk in food allergic children, who are already at risk of nutritional deficiencies and poor growth due to underlying food allergy [30, 31]. Further complicating this picture, there are exemptions to labelling legislation in Canada [32].

Our results provide a Canadian-specific context to an international call by allergists and food allergy activists for a better approach to labelling standards to allow food allergic children to remain safe while enjoying the broadest diet possible [27, 29, 33, 34].

A diagnosis of food allergy has a tremendous impact on food allergic families, as noted by the families in this study. Canadian households spend close to $230 million annually on EAI [35]. Elsewhere, the most significant costs were due to the cost of special foods and loss of annual opportunity costs to the caregivers [36, 37]. Food allergy costs American families$20.5 billion annually, including lost labour productivity, out-of-pocket expenses, and opportunity costs [38]. In Sweden, which has a healthcare system similar to that of Canada [39], families with children allergic to common foods were found to have excess annual costs equivalent to 6100 Canadian dollars (CAD) per child and 7400 CAD per adolescent, compared to age- and sex-matched controls [37]. However, whereas there are tax credits in Canada for other disabilities, including celiac disease, none exist at this time for food allergy [40]. Access to basic care requires patience, as wait times for allergists are long [41], and access to diagnostic methods in the allergy clinic such as OFC can be limited [41, 42]. Funding is also required to improve access to allergists, and allergy therapies.

In the past several years, there has been a surge of information both on the role of early introduction of allergenic solids to prevent food allergy [43–47] as well as the possible role that oral immunotherapy (OIT) may play in a child with food allergies, in particular peanut allergy, in modifying its natural course [48, 49]. Access to basic allergy care, as discussed above, is likely to become increasingly important as OIT moves from research to management. Yet, our understanding of the long-term outcomes of such information, as well as the translation from research to practice, demands considerable resources. As noted by one parent in the study: “*there’s not enough push and money being put into the future of allergies.”* In addition, knowledge gaps exist around guidance about allergy prevention at the primary care level [41, 50]. Knowledge translation efforts, educational tools, and funding are required to ensure that food allergy is prevented when possible, and that access to therapies that may modulate the natural course of food allergy are available if needed.

As with all qualitative studies, we acknowledge that our results are not generalizable [18]. However, qualitative findings are transferable to similar situations. We also acknowledge that most families in our study reported having private or group insurance, thus raising the possibility that these were middle-income families. Whether low-income families would perceive similar food allergy-related policy needs remains unknown. Rates of food allergy amongst low-income Canadians are believed to be lower than amongst middle-income families [51]. Yet, for those low-income families directly affected by food allergy, associated costs are likely to be disproportionately higher to net income than would be seen for middle-income families, whereas health literacy and access to care are likely to be compromised.

The strengths of our study include a description of practical policy desires that would benefit families directly affected by food allergy. Yet, with the exception of more research dollars directed toward food allergy, many of these desires are modest in costs. In addition, our study population included families of children with pediatric allergist-diagnosed with food allergy several years prior to participating in this study. Thus, parents will have had time to live with the daily needs of food allergy, and to consider which policies would best benefit them.

## Conclusions

In summary, food allergy remains a daily burden for affected families, and which ought to be addressed through national policies that more accurately label both priority and non-priority allergens, improve the process for precautionary allergen labeling, improve access to basic allergy care, and support the translation of knowledge from research to standard clinical care.

## Supplementary information

**Additional file 1.** Content questions from the interview guide

## Data Availability

The transcripts generated and analysed during the current study are not publicly available due as they contain information which may lead to the identification participants and their families.

## Abbreviations

CAD: Canadian dollars
EAI: Epinephrine autoinjector
IgE: Immunoglobulin E
OFC: Oral food challenge
OIT: Oral immunotherapy
PAL: Precautionary allergen labelling
VITAL®: Voluntary Incidental Trace Allergen Labelling
