# Supplementary material for: “I want to really crack this nut”: an analysis of parent-perceived policy needs surrounding food allergy
Source: BMC Public Health. 2020 Aug 1;20:1194. doi: 10.1186/s12889-020-09309-w (PMC7395383; doi:10.1186/s12889-020-09309-w)
Supplement: Supplementary file 1 — Additional file 1. Content questions from the interview guide [file 12889_2020_9309_MOESM1_ESM.docx]

**Additional File 1.** Content questions from the interview guide

| What would you like schools to know about food allergy? |
| --- |
| What would you like healthcare professionals to know about food allergy? |
| What would you like policy makers to know about food allergy? |
